# Two-Component Systems Are Involved in the Regulation of Botulinum Neurotoxin Synthesis in *Clostridium botulinum* Type A Strain Hall

**DOI:** 10.1371/journal.pone.0041848

**Published:** 2012-07-26

**Authors:** Chloé Connan, Holger Brueggemann, Christelle Mazuet, Stéphanie Raffestin, Nadège Cayet, Michel R. Popoff

**Affiliations:** 1 Institut Pasteur, Bactéries anaérobies et Toxines, Paris, France; 2 Department of Biomedicine, Aarhus University, Aarhus, Denmark; 3 Institut Pasteur, Plateforme de Microscopie Ultrastructurale, Paris, France; University of Padova, Italy

## Abstract

*Clostridium botulinum* synthesizes a potent neurotoxin (BoNT) which associates with non-toxic proteins (ANTPs) to form complexes of various sizes. The *bont* and *antp* genes are clustered in two operons. In *C. botulinum* type A, *bont/A* and *antp* genes are expressed during the end of the exponential growth phase and the beginning of the stationary phase under the control of an alternative sigma factor encoded by *botR/A*, which is located between the two operons. In the genome of *C. botulinum* type A strain Hall, 30 gene pairs predicted to encode two-component systems (TCSs) and 9 orphan regulatory genes have been identified. Therefore, 34 Hall isogenic antisense strains on predicted regulatory genes (29 TCSs and 5 orphan regulatory genes) have been obtained by a mRNA antisense procedure. Two TCS isogenic antisense strains showed more rapid growth kinetics and reduced BoNT/A production than the control strain, as well as increased bacterial lysis and impairment of the bacterial cell wall structure. Three other TCS isogenic antisense strains induced a low level of BoNT/A and ANTP production. Interestingly, reduced expression of *bont/A* and *antp* genes was shown to be independent of *botR/A*. These results indicate that BoNT/A synthesis is under the control of a complex network of regulation including directly at least three TCSs.

## Introduction

Botulinum neurotoxins (BoNTs) are the most potent toxins known, and seven BoNT toxinotypes (A to G) have been recognized based upon their antigenic properties. They are produced by distinct strains of *Clostridium botulinum* and by atypical strains of other *Clostridium* species that display heterogeneous bacteriological characteristics. BoNTs are synthesized as single chain proteins (ap. 150 kDa) and exported outside of the bacteria by an unknown mechanism. Each toxinotype is proteolytically cleaved into a heavy chain (H, ap. 100 kDa) and light chain (L, ap. 50 kDa), which remain linked by a disulfide bridge. The H chain recognizes through its C-terminal part (Hc) a specific cell surface receptor on nerve endings, and then facilitates toxin uptake into cells by receptor-mediated endocytosis. The L chain (Lc) translocates into the cytosol of motoneurons where they gain access to substrate. L chains are zinc dependent proteases which specifically cleave one of the three soluble *N*-ethylmaleimide sensitive factor (NSF) attachment protein receptors (SNARE), thus blocking evoked acetylcholine release at the skeletal neuromuscular junction [1], [2].

BoNTs and associated non-toxic proteins (ANTPs) form complexes of various sizes (300 to 900 kDa). ANTPs consist of a non-toxic non-hemagglutinin component (NTNH), similar in size to BoNT, hemagglutinin components (HAs) or other non-hemaggltuinin proteins called OrfX1, OrfX2, OrfX3 or P47 [3]–[6]. ANTPs spontaneously associate with BoNT at low pH by non covalent bonds and form stable complexes that dissociate at high pH (pH 8 and above) (rev in [4]). ANTPs are involved in BoNT protection against acidic pH and proteolytic degradation during passage through the stomach and intestine. In addition, HA33/35 and HA50, which bind to distinct carbohydrate structures, have been found to facilitate BoNT transport across the intestinal barrier [7], [8]. It has been reported that HAs of type A and type B, but not type C, bind to the extracellular domain of human epithelial E-cadherin and increase the paracellular permeability of the intestinal barrier by a yet unknown mechanism [9].

The *bont* and *antp* genes are clustered in close vicinity and constitute the botulinum locus. According to *bont* sequence and botulinum locus gene organization each botulinum type is divided in subtypes [10]. In *C. botulinum* subtype A1 strain Hall, *bont* and *antp* genes are organized in two operons. The first operon (*ntnh/A-bont/A*), which is located at the 3′ end of the *botulinum* locus, encompasses the *bont* gene immediately preceded by the *ntnh/A* gene. Both genes are transcribed in the same orientation, and the organization of this operon is highly conserved in all *C. botulinum* types. The second operon contains the *ha* genes and differs slightly between various toxinotypes. In strain Hall, the *ha* operon contains successive genes for the 34 kDa (*ha34*), 17 kDa (*ha*17), and 70 kDa (*ha*70) HAs [11].

The *botR/A* gene is localized between the two operons in strain Hall and is transcribed in the same orientation than BoNT/A. BotR/A has been characterized as an alternative sigma factor of RNA polymerase, which positively regulates the transcription of *bont* and *antp* genes [12], [13]. BotR/A forms with *Clostridium tetani* TetR, *C. difficile* TxeR (or TcdR) and *Clostridium perfringens* UviA, a new sub-group of the sigma 70 family of RNA polymerase involved in controlling clostridial toxin genes [13]. Transcription of *bont* and *antp* genes in strain Hall is highly regulated at the end of the exponential growth phase and beginning of the stationary phase [14]. This supposes that a complex regulatory network may be involved in the control of toxin synthesis in *C. botulinum*. Bacteria typically sense environmental changes using a phosphorelay system including at least two proteins known as two-component systems (TCSs). TCSs consist in a sensor histidine kinase (SHK), which detects a signal and autophosphorylates on a histidyl residue. The phosphate group is then transferred to a conserved aspartyl residue of the response regulator (RR) protein which is typically a DNA-binding protein that regulates target gene expression [15]. Some TCSs are known to be involved in the regulation of virulence factors in Gram-negative and Gram-positive bacteria. Sequencing of *C. botulinum* genomes has identified numerous potential regulatory systems, including TCSs. According to Sebaihai et al. *C. botulinum* strain Hall genome contains 28 putative TCSs, 8 orphan histidine kinases, 8 orphan response regulators, and 15 sigma factors [11]. In this study we investigated the potential role of 34 regulatory genes in the regulation of toxin synthesis; isogenic antisense strains were constructed using a mRNA antisense approach to silence the response regulator genes.

## Results

### Analysis of *botR/A* isogenic antisense strains

**Figure 1:**
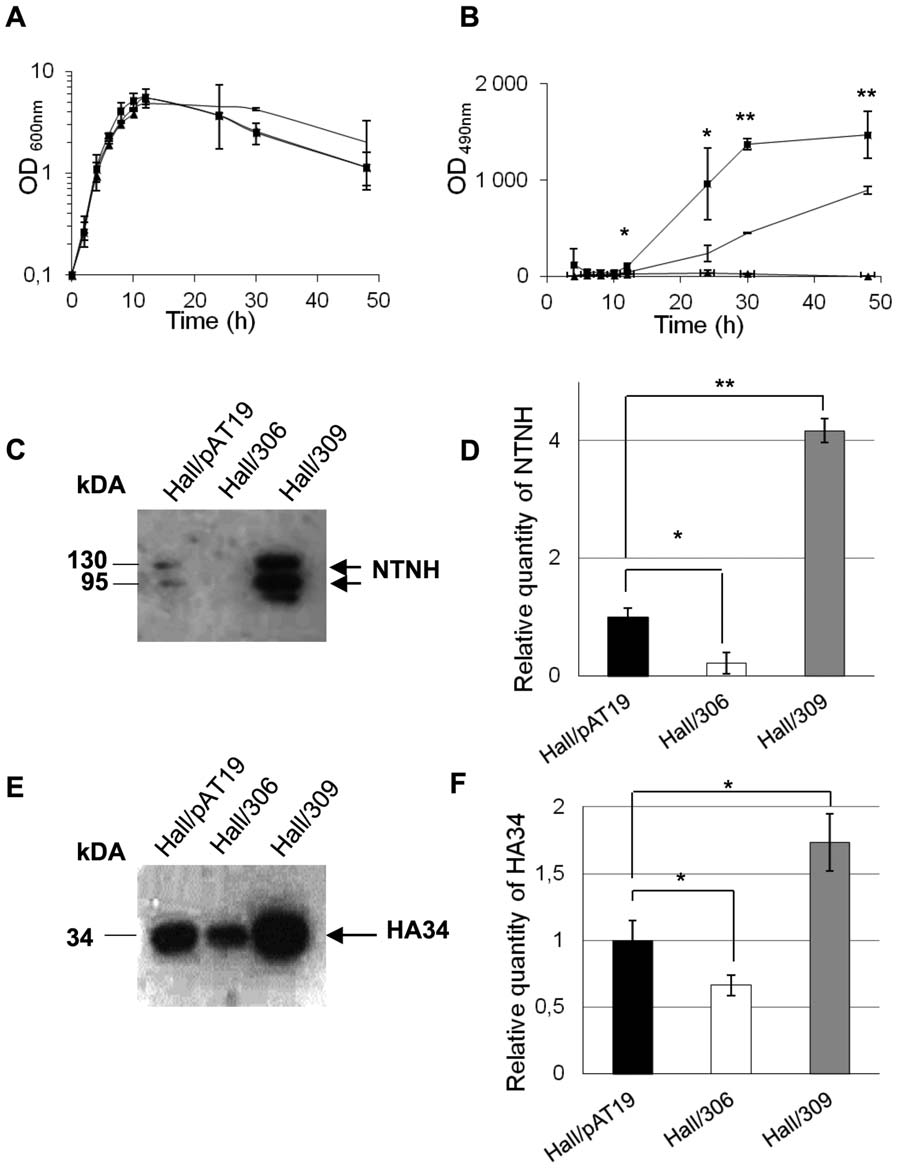
Growth kinetics, BoNT/A, NTNH, and HA34 production in Hall *botR/A* isogenic antisense strains. (A) Growth curves of Hall/pAT19 (empty vector), Hall/306 (antisense *botR/A* mRNA), and Hall/309 (*botR/A* overexpression) were determined by measurement of optical density at 600 nm (OD_600_). (B) BoNT/A was assayed in the culture supernatants by ELISA and expressed as OD at 490 nm. Production of NTNH (C) and HA34 (E) were determined in the supernatant of 12 h cultures by Western blotting with specific antibodies and quantification with ImageJ (D, F) (means +/− SD; n = 3), *P<0.05 and **P<0.005. Note that western blotting with anti-NTNH antibodies show native (130 kDa) and partially processed NTNH in its 115 kDa fragment.

Investigation of putative regulatory genes of the toxinogenesis in *C. botulinum* Hall was performed with the anti-sense mRNA methodology developed for the study of *botR/A* with some modifications [12]. Partial inhibition of *botR/A* expression was obtained by transfection of Hall strain with the plasmid pMRP306, which contains the 5′end of *botR/A* gene and the ribosome binding site (RBS) region cloned in opposite direction downstream of the iota toxin gene promoter in the pAT19 vector [12]. In contrast, transfection with pMRP309, which harbors the whole *botR/A* gene under its own promoter in pAT19 vector, resulted in an overexpression of *botR/A*. Strain Hall/pAT19 contains the empty pAT19 vector. Cultures of strains Hall/306, and Hall/309 strains showed similar growth kinetics in TGY medium compared to the control strain Hall/pAT19 (Fig. 1A) or wild type strain Hall (data not shown) indicating that the vector alone or containing the anti-sense construct did not significantly modify the main metabolism of *C. botulinum* Hall. As expected, BoNT/A detected in the culture supernatant with ELISA, was drastically repressed in Hall/306 and overproduced in Hall/309 at 24, 30 and 48 h of growth compared to Hall/pAT19 as control (Fig. 1B). The effects of partial repression or overexpression of *botR/A* on ANTP production were checked by quantified western blotting with anti-NTNH and anti-HA34 antibodies. Partial inhibition of *botR/A* induced a significantly lower production of NTNH and HA34, whereas opposite effects were observed when *botR/A* was overexpressed (Fig. 1C–F).

**Figure 2:**
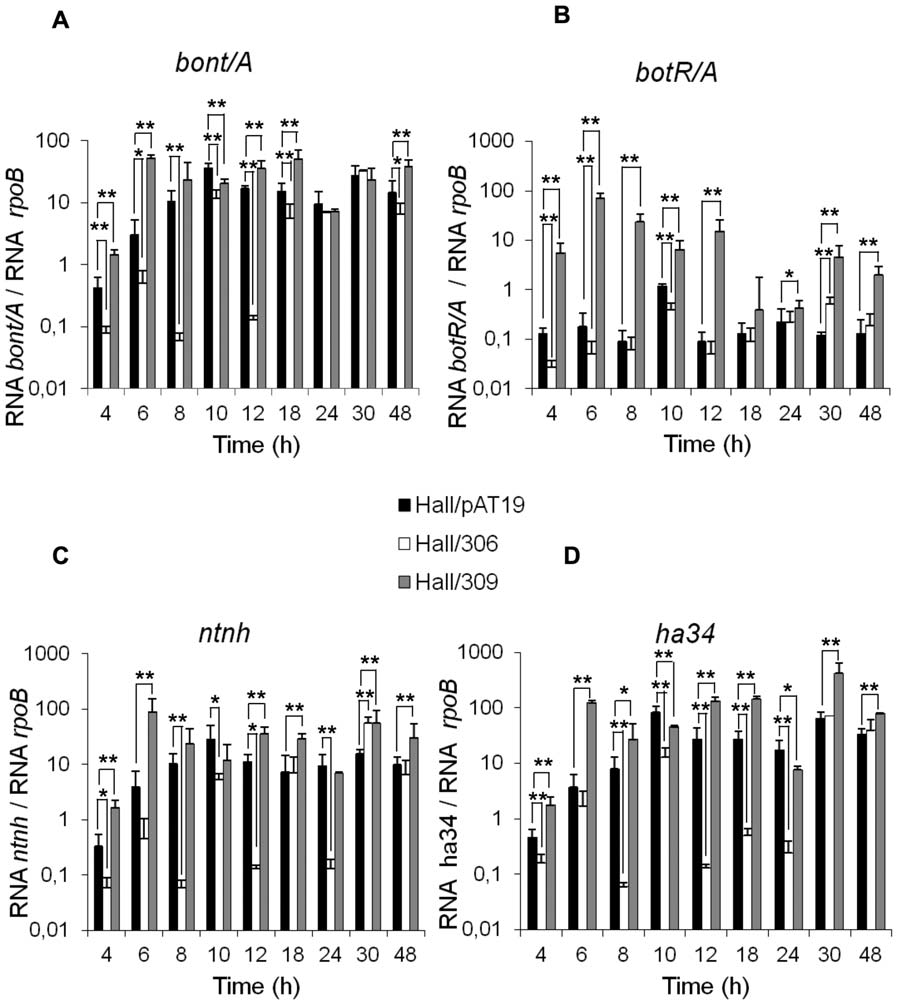
Kinetics of transcript levels of *bont/A, botR/A, ntnh*, and *ha34* genes in Hall *botR/A* isogenic antisense strains. Transcript levels were quantified by qRT-PCR at 4, 6, 8, 10, 12, 18, 24, 30 and 48 h of culture and normalized versus housekeeping rpoB gene in Hall/pAT19 (empty vector), Hall/306 (antisense *botR/A* mRNA), and Hall/309 (*botR/A* overexpression). Data are mean values +/− SD from 9 independent experiments, *P<0.05 and **P<0.005.

Expression of the target genes was monitored by quantitative reverse transcriptase PCR (qRT-PCR) with the primers listed in Table 1; normalization was based on the expression of the house keeping gene *rpoB*. As shown in Fig. 2, *bont/A, ntnh*, and *ha34* genes were significantly repressed in Hall/306, mainly from the mid exponential growth phase (4–8 h) until the early stationary phase (18–24 h). Opposite effects were observed when *botR/A* was overexpressed. However, the effects of *botR/A* overexpression on *bont/A, ntnh*, and *ha34* genes were only significant during the exponential growth phase (4–12 h). As monitored by qRT-PCR, *botR/A* was markedly overexpressed during the exponential growth phase (4–12 h) of Hall/309 and to a lower extent during the stationary phase, whereas a decrease in *botR/A* expression was observed only within the exponential growth phase of Hall/306 (Fig. 2B).

**Table 1:** Primers used to amplify internal fragments of the target genes by quantitative reverse transcriptase PCR.

Gene	Primer*	Séquence (5′-3′)	Product length (bp)	*T_m_* (°C)	PCR efficiency (%)
*rpoB*	F	TTCCTCCATCGCTATCTCGT	175	57.5°C	105.1
	R	AAACATGCAACGTCAAGCAG			
*bont/A*	F	AATGGTTATGGCTCTACTCAATAC	134	52.7°C	99.6
	R	GCTAATGTTACTGCTGGATCTG			
*botR/A*	F	TGACAACGAGGAGTTTCAAGA	146	55.1°C	95.6
	R	TGAAATTGCTCAAATCAACTTTTT			
*ha34*	F	AAGTCGTACAACAAGTGGATATG	148	53°C	105.3
	R	GTTAGAACTCCGTTTGAAAGTATTG			
*ntnh/A*	F	AATGTTGTAGTAGTTAGAGCTAG	107	45.1°C	96.5
	R	CTCAAAGATTCGCCATAATATC			
CLC_1093	F	CTGCAATGGATGGTGAAGAA	912	60.1°C	89.10
	R	CTTGCCACAAGCTCCCTTAC			
CLC_1094	F	GATGGATTAATTGCGGTGGA	865	60.5°C	106.40
	R	TCATCTCATCCTCCTCTTCTCC			
CLC_0661	F	AGCTAAAGATGCAAAAGATGCT	889	58.7°C	95.30
	R	CACAATATCGTTACGGGCAGT			

PCR efficiencies correspond to the optimized PCR parameters indicated in Materials and Methods (see also Table S1).

F, forward; R, reverse.

### Construction of TCS isogenic antisense strains

In further analyses we searched the genome of *C. botulinum* strain Hall for additional regulatory systems, in particular TCSs. 39 proteins could be considered as RRs: they all possessed the signal receiver domain Rec (cd09944) and an additional domain, usually the DNA binding domain HTH_XRE (cl00088) or LytTR (cl04498). This is in agreement with the recent analysis of Galperin et al. [16] showing that bacterial signal transduction proteins retain a conserved protein family profile. Indeed, of the 39 RRs that we have identified in the Hall strain genome, the majority (26) belongs to the OmpR family (COG0745) consisting of RRs with a CheY-like receiver domain and a winged-helix DNA-binding domain, and 4 other RRs were LytR family members (COG3279). Nine RRs were orphan regulators and 30 RR genes were located next to a gene coding for a SHK, as judged by the presence of two domains in the respective gene product, a histidine kinase-like ATPase (HATPase_c, cd00075) and a histidine kinase A (dimerization/phosphoacceptor) domain (HisKA, cl00080). Most TCSs of *C. botulinum* strain Hall have close homologues in other *Clostridia*. Besides *C. sporogenes*, which is phenotypically related to *C. botulinum* but non-toxigenic, homologs TCSs exist in particular in *C. carboxidivorans*. However, 12 RRs have low similarity to proteins in other clostridia. Two RRs (CLC_0632 and CLC_1105) show homology (38 and 37% protein identity, respectively) to the VirR regulator, which is part of the *Clostridium perfringens* TCS VirR-VirS involved in the regulation of numerous toxins in this bacterium [17]–[24].

Plasmids able to generate anti-sense mRNA from 34 predicted regulatory genes were constructed similarly to pMRP306 and transfected into strain Hall. DNA segments for anti-sense mRNA production were designed in the RR gene of 25 TCSs, in the SHK gene in 4 other TCSs, as well as in 5 orphan regulatory genes (Table 2).

**Table 2:** Two-component system and orphan response regulator genes in *C. botulinum* strain Hall, homology with regulatory genes in other bacterial species, Hall isogenic antisense strains, and primers used for the construction of recombinant plasmids generating antisense mRNA.

Iosgenic antisense strains	Gene Bank accession number^a^	family (RR)	Homologs (RR) in other clostridia^b^	Nucleotide sequence (5′→3′)
Hall/651	**RR CLC_1229 (SHK CLC_1230)**	LytR	*C. ljungdahlii*	CCGCTGCAGGGAAAAAATGTTGTTAGTATTTTTCATG CCCCATGGTCATTATCCTCACAAATCATTACTC
Hall/652	**SHK CLC_0669 (RR CLC_0668)**	YcbB domain	*/*	CCGCTGCAGAAGCTTGATAAGGAGATGAAAATAATG GGCCATGGATACCAAAAGATATATTGAAATGATTAAC
Hall/653	**SHK CLC_3251 (RR CLC_3250)**	LytR	*/*	CCGCTGCAGGAGAAATTTATGATATATATACAATTATTAG CCCCATGGCAGATTTGTTTTTAATAAATTTATTAAAAG
Hall/660	**RR CLC_1431 (SHK CLC_1432)**	OmpR	*/*	CCGCTGCAGCAGGAGTGATATATATGAGTTATAAG GGCCATGGCTACTAGTGCTTCGTATCC
Hall/661	**RR CLC_2236 (SHK CLC_2235)**	OmpR	*C. sp. 7_2_43FAA C. perfringens C. beijerinckii C. sp. L2-50 C. cellulovorans C. ljungdahlii*	CCGCTGCAGGCGGATAAGAATGGTTAAGAT GGCCATGGCCCGTTCTACCATTATTTGCTTTTAC
Hall/662	**Orphan RR CLC_2624**	CheB	*C. carboxidivorans C. novyi NT C. ljungdahlii C. kluyveri*	CCGCTGCAGGAGTGATTCATTTGAATAAAATTAAAG GGCCATGGCTGTATCTATTACTTCTAAATCATTTTC
Hall/663	**RR CLC_0376 (SHK CLC_ 0377)**	OmpR	*C. carboxidivorans C. difficile*	CCGCTGCAGCCTATAATGAATAAGGAGAAAATG GGCCATGGCTATCTTTACATCAAATCCATATTTAG
Hall/664	**RR CLC_2386 (SHK CLC_2385)**	OmpR	*C. kluyveri C. acetobutylicum C. ljungdahlii C. carboxidivorans C. tetani C. butyricum C. cellulovorans C. sp. 7_2_43FAA C. beijerinckii C. perfringens*	CCGCTGCAGGAGGGGTACCATGGCTATGGAA GGCCATGGCCCATTCAAAGCACAAATACAGTTATAAC
Hall/666	**RR CLC_1871 (SHK CLC_1870)**	OmpR	*/*	CCGCTGCAGAGGTGTTTATATGCAGGAAAA GGCCATGGCTTCTCTACCATTACTAGCTGTAATAAC
Hall/667	**RR CLC_0842 (SHK CLC_0843)**	OmpR	*/*	CCGCTGCAGGGGGATTTTGTGACAAAAATATTATTAG GGCCATGGCTCTTTTAACTTCATAATTTTCTTGTT
Hall/673	**SHK CLC_0306 (RR CLC_0307)**	NarL	*C. butyricum*	CCGCTGCAGGTGAGAATATGAGAAAAGCAATGAAGC GGCCATGGTTTCCAGTCATCCATGAAACAACAAAAG
Hall/706	**RR CLC_1640 (SHK CLC_1639)**	OmpR	*C. carboxidivorans C. novyi NT C. tetani C. bartlettii C. difficile C. papyrosolvens*	CCGCTGCAGAAAGGATGGGACAAGTATGATTG GGCCATGGCCATCTTTAGCTTTAAAAACAGTTATACC
Hall/707	**RR CLC_1093 (SHK CLC_1094)**	OmpR	*C. carboxidivorans C. beijerinckii*	CCGCTGCAGGGAGAGAAGATATGAAGAAACTC GGCCATGGCCATCCATTGCAGTTATCACTTCATAATC
Hall707k	**SHK CLC_1094 (RR CLC_1093)**			CCGCTGCAGATTTGGAGGAAAAAATTAATG GGCCATGGATCCTCTTTATACATATCTTC
Hall/708	**RR CLC_0423 (SHK CLC_0424)**	OmpR	*C. carboxidivorans C. tetani C. acetobutylicum C. ljungdahlii C. sp. 7_2_43FAA C. butyricum C. cellulovorans C. beijerinckii*	CCGCTGCAGGAGATGATTAAGAAATGTCAGCAG GGCCATGGATGCCTTTAAAGTTATATAACCTTCATTG
Hall/714	**RR CLC_1914 (SHK CLC_1913)**	OmpR	*/*	CCGCTGCAGGAGATGGATTACAATGCCAAAGAG GGCCATGGCCACCTCCACTAAATATCCACTTTCC
Hall714k	**SHK CLC_1913 (RR CLC_1914**)			CCGCTGCAGTAAGGGGAGTGGGGTATAAAA GGCCATGGATTTATATCTAATTTAGG
Hall/716	**RR CLC_2079 (SHK CLC_2078)**	OmpR	*C. ljungdahlii C. carboxidivorans C. beijerinckii C. bartlettii C. butyricum C. perfringens C. sp. 7_2_43FAA*	CCGCTGCAGGGAGGTAGAGTAAGTAATGAAAAAAATTC GGCCATGGCTCTACAGGTGCATTTACTTCGTATCC
Hall/717	**RR CLC_1415 (SHK CLC_1414)**	NarL	*C. acetobutylicum C. sp. 7_2_43FAA C. difficile*	CCGCTGCAGGGGAAAAATAAAATGAATAAAACTAATA GGCCATGGCCGGCAAGACCTACAACTTCAATATCATTG
Hall/720	**RR CLC_0354 (SHK CLC_0355)**	OmpR	*C. acetobutylicum C. kluyveri*	CCGCTGCAGGGGTGATAAATATGGGAAGAAAAGTC GGCCATGGTTACTTCATACCCTTCACGTTGTAGATAC
Hall/722	**RR CLC_3521 (SHK CLC_3520)**	OmpR	*C. carboxidivorans C kluyveri C. novyi NT C. ljungdahlii C. tetani C. sp. 7_2_43FAA C. beijerinckii C. butyricum C. acetobutylicum C. perfringens C. cellulovorans C. thermocellum*	CCGCTGCAGGAGGATATACCAATGGAAGG GGCCATGGTGTCGTATCCTGAATTTTCCAAATAC
Hall/723	**RR CLC_1024 (SHK CLC_1025)**	OmpR	*C. phytofermentans C. difficile*	CCGCTGCAGAGGGATTAATATGGATGCAATATCTTT GGCCATGGCCTTCTTTTTTAAGTACTGTTCTTATTAATC
Hall/724	**RR CLC_1088 (SHK CLC_1089)**	OmpR	*C. sp. 7_2_43FAA C. carboxidivorans C. acetobutylicum C. ljungdahlii C. beijerinckii C. tetani C. cellulovorans*	CCGCTGCAGAAGGTGAAATAAATGAGTAACATTTTAAT GGCCATGGTAGTGCAATATACTTTATATCCTTCTTTC
Hall/1001	**Orphan RR CLC_0632**	LytR	*/*	CTGCAGAACTAATAAAAGGGAGGACAT CCATGGCTTTAATAATTTTTATTAGAT
Hall/1002	**RR CLC_1105 (SHK CLC_1104)**	LytR	*C. carboxidivorans C. ljungdahlii*	CTGCAGTACTTACTTTGGAGGTGATTC CCATGGTTTTTTAATCGTAAAGCTTC
Hall/1003	**RR CLC_0665 (SHK CLC_0666)**	OmpR	*C. sp. 7_2_43FAA C. beijerinckii C. difficile C. bartlettii*	CTGCAGATATAATTGGAGGAAAATATG CCATGGTTAAACACATTATACCCCTCA
Hall/1004	**RR CLC_0331 (SHK CLC_0232)**	OmpR	*C. tetani C. acetobutylicum C. carboxidivorans C. ljungdahlii*	CTGCAGTATTTAGAGGAGATTTTTCTA CCATGGTATACAAAATTATCACAACC
Hall/1140	**RR CLC_0323 (SHK CLC_0324)**	OmpR	*/*	CCGCTGCAGGTACCTATATGGAGGTATAATTTTATG GGCCATGGCCTCATTTTCTAAATACAATTTTATAAC
Hall/1141	**Orphan RR CLC_0958**	OmpR	*C. cellulovorans C. beijerinckii C. sp. M62/1 C. symbiosum*	CCGCTGCAGAACCTATTAAAAAGGAGGTCTAGATAATG GGCCATGGCCATTTTTATTAAGTACTGTAC
Hall/1142	**RR CLC_2212 (SHK CLC_2211)**	OmpR	*/*	CCGCTGCAGATATAAGGTGATATTATGGAAAGG GGCCATGGCGTATCCTTCTTTTATCAAGG
Hall/1143	**Orphan RR CLC_0577**	WspR	*/*	CCGCTGCAGTTAAATATGAGAGTGATAAGCGTGC GGCCATGGTTTATGTCTTTTTCCTCTAAAAGATCo
Hall/1144	**Orphan RR CLC_0580**	Pas_4 and Hpt domains	*/*	CGCTGCAGTAGGGAAGGATAAAATTTATGGTAAAG GGCCATGGCTTTATCCAAAATGAATATGGAAACG
Hall/1145	**SHK CLC_1867 (RR CLC_1866)**	OmpR	*C. beijerinckii C. bartlettii C. butyricum C. novyi C. tetani*	CCGCTGCAGATGGGGGGGAATTAGTTTGCG GGCCATGGGCTTTCTATACTTCTTAAATATATTTC
Hall/1146	**RR CLC_0661 (SHK CLC_0663)**	OmpR	*C. tetani C. carboxidivorans C. sp. 7_2_43FAA*	CCGCTGCAGAAGCAATAGGAGGAATTTATAATGG GGCCATGGATCCATCCGCTCTTAGAGCAG
Hall1146k	**SHK CLC_0663 (RR CLC_0661)**			CCGCTGCAGAGGAATTCAGAAGCATAAC
GGCCATGGTAACTATAAATGTTCC				
Hall/1147	**RR CLC_0410 (SHK CLC_0411)**	OmpR	*C. perfringens C. sp. 7_2_43FAA C. ljungdahlii*	CCGCTGCAGTGAGGTGAAGCCATGG GGCCATGGCTGCTTCTATTACATCAAAATTGTTCATC
Hall/1148	**RR CLC_3294 (SHK CLC_3293)**	OmpR	*/*	CCGCTGCAGAGGGGGAGTGCTAATTGAG GGCCATGGACATCATATCCTTCTTTAAT

a In bold the accession number of the gene which has been targeted in the isogenic antisense strains and in bracket the associated gene of the corresponding TCS.

b Protein identity >60% (*C. sporogenes* is omitted due to its high similarity to *C. botulinum*).

**Figure 3:**
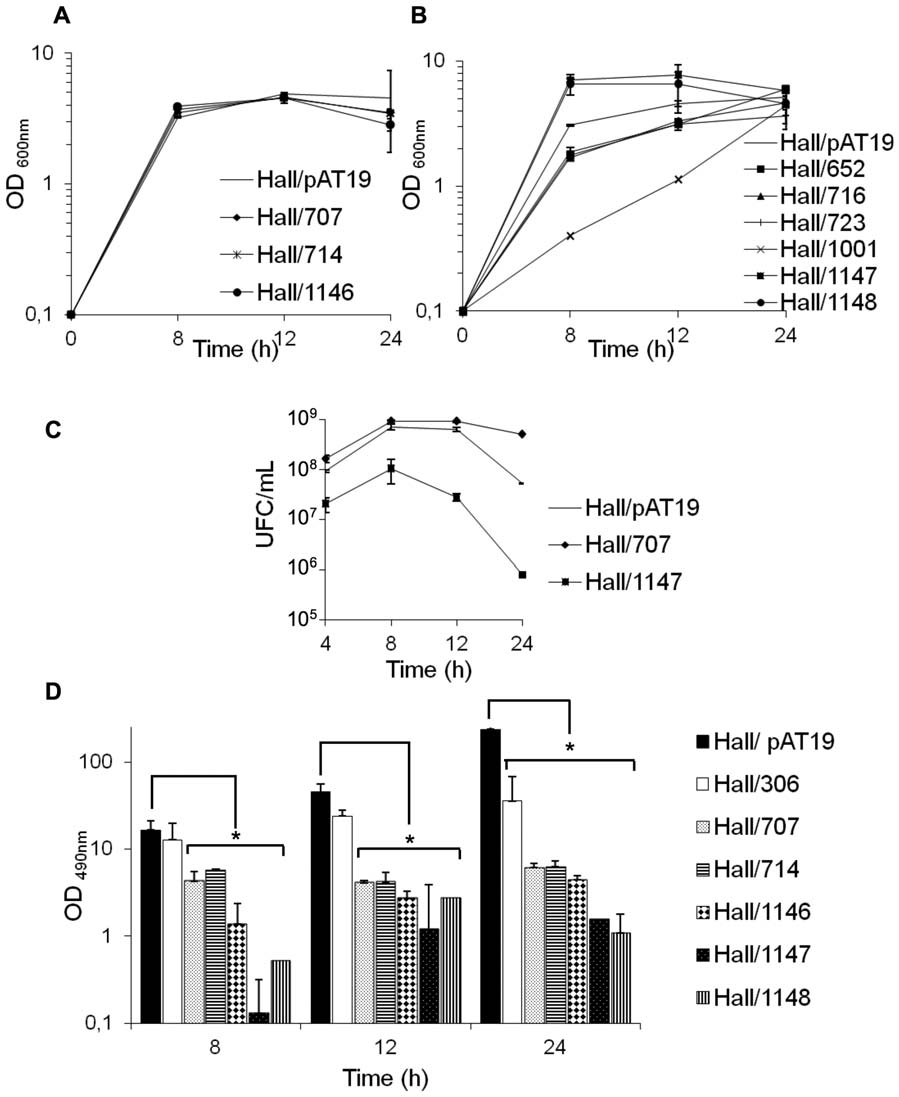
Growth kinetics and BoNT/A production in representative Hall isogenic antisense strains. (A) Hall/707, Hall/714, and Hall/1146 showed a similar growth curve monitored at OD_600_ than the Hall/pAT19 strain. (B) Hall/1147 and Hall/1148 grew more rapidly than the control Hall/pAT19 strain, whereas Hall/1001 displayed a slower growth curve. Hall/652, Hall716, and Hall/723 show a similar kinetics compare to Hall/pAT19. (C) Monitoring of growth kinetics of Hall/pAT19, Hall/707 and Hall/1147 by bacterial counting on TGY agarose and expressed as unit forming colony (UFC)/ml. (D) BoNT/A production assayed by ELISA was significantly reduced in the culture supernatants of Hall/707, Hall/714, Hall/1146, Hall/1147, and Hall/748, versus the control Hall/pAT19 strain. BoNT/A production was significantly reduced in Hall/306 at 24 h culture. Data are mean values +/− SD from 3 independent experiments, *P<0.05.

Growth kinetics of Hall isogenic antisense strains in TGY broth medium supplemented with erythromycin (10 µg/ml) were monitored by spectrometry at 600 nm over a 24 h period. 32 Hall isogenic antisense strains showed similar growth kinetics compared to the control strains (Hall/pAT19 and wild type Hall) (Fig. 3A, 3B and data not shown). However, growth of strain Hall/1001 was drastically delayed, and to a lesser extent that of Hall/652, Hall/716 and Hall/723, whereas two other isogenic antisense strains (Hall/1147 and Hall/1148) grew faster than the control Hall/pAT19 (Fig. 3B). However, the five isogenic antisense strains reached a similar OD than the other isogenic antisense strains at 24 h. Cell counts were performed with three representative strains. Hall/pAT19 and Hall/707 showed similar growth kinetics than those monitored by OD_600 nm_ measurements, which were characterized by an exponential growth curve reaching a plateau at 8–12 h followed by a slow decrease in viability (Fig. 3C). However, Hall/1147 exhibited a different pattern characterized by a growth curve plateau at 8 h, which was slightly lower than that of Hall/pAT19 and which was followed by a rapid and drastic decrease in viability (Fig. 3C).

### BoNT/A production is reduced in five TCS isogenic antisense strains

Among the 34 Hall isogenic antisense strains, 5 showed reduced BoNT/A production in the culture supernatant in comparison to Hall/pAT19 as tested by ELISA, namely Hall/707, Hall/714, Hall/1146, Hall/1147 and Hall/1148 (Fig. 3D). The lower levels of BoNT/A in the culture supernatants of these isogenic antisense strains were confirmed by the mouse bioassay, indicating reduced amounts of biologically active toxin (Table 3). A TCS was targeted in these 5 isogenic antisense strains with decreased BoNT/A production. In contrast, the 5 orphan regulatory gene isogenic antisense strains retained the same levels of BoNT/A production than the control strain Hall/pAT19. The five isogenic antisense strains produced a significantly lower level of BoNT/A than Hall/pAT19 and Hall/306 (Fig. 3D). Indeed a 8 to 10 fold decrease of BoNT/A production was measured in the supernatant of these five isogenic antisense strains at 12 and 24 h compared to Hall/306.The strains Hall/1001, Hall/652, Hall/716 and Hall/723 which exhibited a delayed growth kinetic, were also delayed in toxin production but yielded a similar BoNT/A level at 24 h compared to Hall/pAT19 (data not shown). These results indicate that these mutants displayed a delayed BoNT/A synthesis or impaired secretion, but were not affected in the global toxin synthesis rate.

**Table 3:** BoNT/A production tested by mouse bioassay in 12 h culture supernatants of the control strain Hall/pAT19 and isogenic antisense strains.

Recombinant strains	Mouse bioassay (DL100/ml)
Hall/pAT19	2.10^3^
Hall/306	20
Hall/309	2.10^5^
Hall/707 (CLC_1093)	<2
Hall/714 (CLC_1914)	<2
Hall/1146 (CLC_0661)	<2
Hall/1147 (CLC_0410)	<2
Hall/1148 (CLC_3294)	<2

### Two TCS isogenic antisense strains, Hall/1147 and Hall/1148, show an altered bacterial cell wall/surface layer

**Figure 4:**
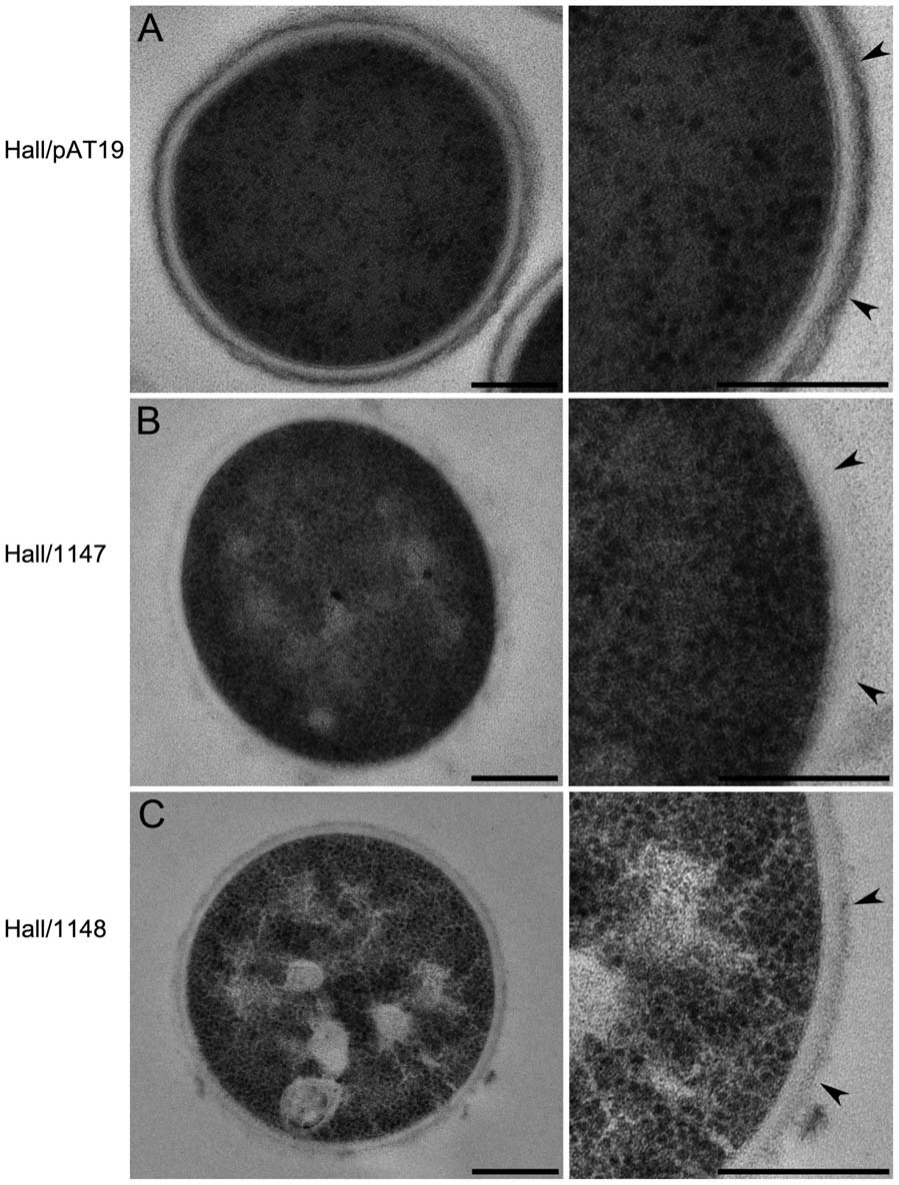
Ultrastructural morphology of Hall/pAT19, Hall/1447, and Hall/1148. Bacteria from a 5 h culture in TGY were treated by high pressure and freeze substitution and then observed by electron microscopy. Hall/pAT19 (A) showed a well delineated wall, whereas in Hall/1147 (B) and Hall/1138 (C) the bacterial wall is transparent, almost invisible. In addition, Hall/1148 (C) showed numerous transparent inclusions. All scale bars represent 200 nm.

Two strains out of the five isogenic antisense strains with a decreased BoNT/A production grew differently compared to control strains. Cultures of the TCS isogenic antisense strains Hall/1147 and Hall/1148, were very viscous at the end of the exponential growth phase, and the OD_600_ was significantly higher than the control strain Hall/pAT19 (Fig. 3B). Cell counting confirmed that Hall/1147 grew rapidly until 8 h similarly to Hall/pAT19, although growth yield of Hall/1147 was one log level reduced compared to the control strain. But Hall/1147 viability decreased rapidly in the stationary growth phase, with over two log differences at 12 h compared to the control strain (Fig. 3B). Hall/1148 showed a similar growth profile than Hall/1147 (data not shown). This growth behavior suggests that Hall/1147 and Hall/1148 lyse readily and early. Therefore, we analyzed the strains by transmission electron microscopy during the mid exponential growth phase (5 h of culture). After high pressure freezing and freeze substitution, the cell wall of the control cells showed a layered organization. The outer part had an electron dense appearance, whereas the inner part next to the plasma membrane was electron lucent (Fig. 4A). The electron dense outer layer was less developed or absent in the Hall/1147 and Hall/1148 strains (Fig. 4B–C). In addition, the cytoplasm of Hall/1148 bacteria was less homogenous compared to the control cells and showed apparently empty areas of various sizes and shapes (Fig. 4C). This indicates that the TCSs, which were partially repressed in Hall/1147 and Hall/1148, induced pleiotropic effects including the regulation of bacterial cell wall or cell surface synthesis as well as toxin synthesis as shown in Fig. 3C.

### Three TCSs control *botR/A, bont/A* and *antp* expression at the transcriptional level

Three TCS isogenic antisense strains (Hall/707, Hall/714, Hall/1146) showed reduced BoNT/A levels during the exponential and stationary growth phases, similarly to the isogenic antisense strain repressed in *botR/A* (i.e. Hall/306) (Fig. 3B) No increase in culture viscosity and no microscopic alterations of the bacterial cell wall was observed in these three strains (data not shown).

**Figure 5:**
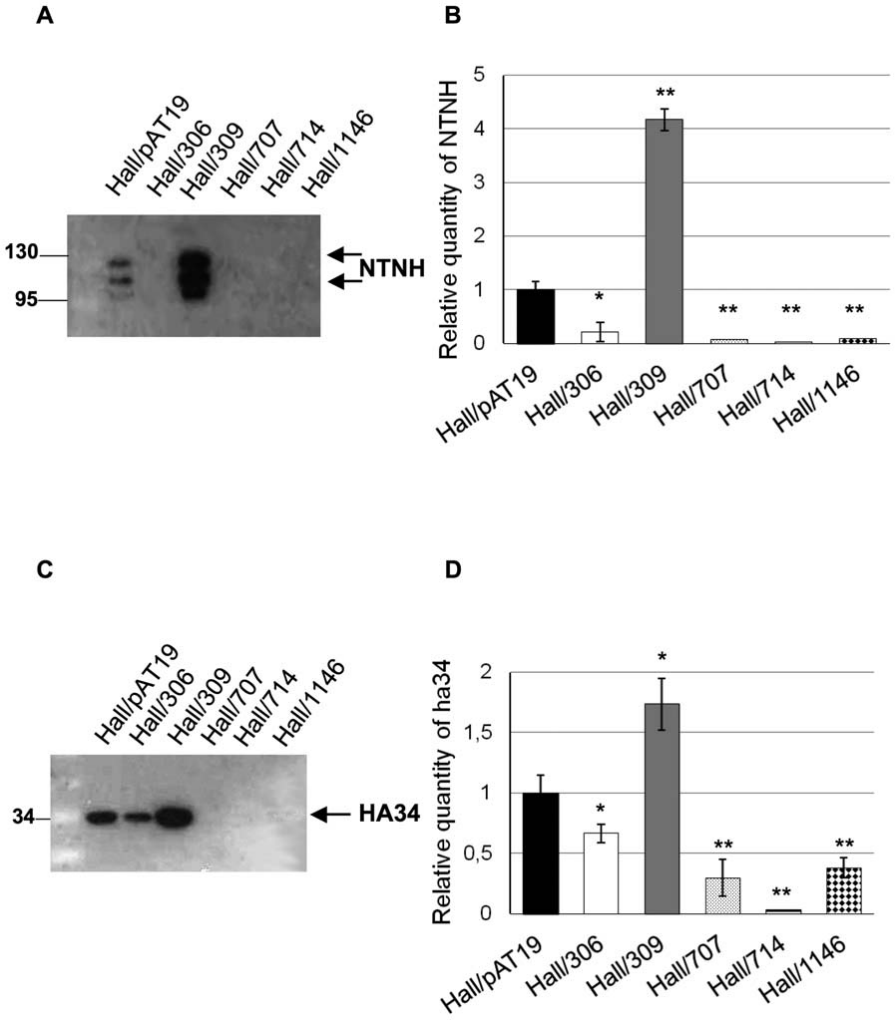
NTNH and HA34 production in Hall *botR/A* and 3 TCS isogenic antisense strains. Production of NTNH (A) and HA34 (B) was assayed by western blotting in 12 h culture supernatants of Hall/pAT19, Hall/306, Hall/309, Hall/707, Hall714, and Hall/1146. Western blots were quantified by ImageJ (B and D) with normalization of NTNH and HA34 production in Hall/pAT19 to 1. Data are mean values +/− SD from 3 independent experiments, *P<0.05 and **P<0.005.

**Figure 6:**
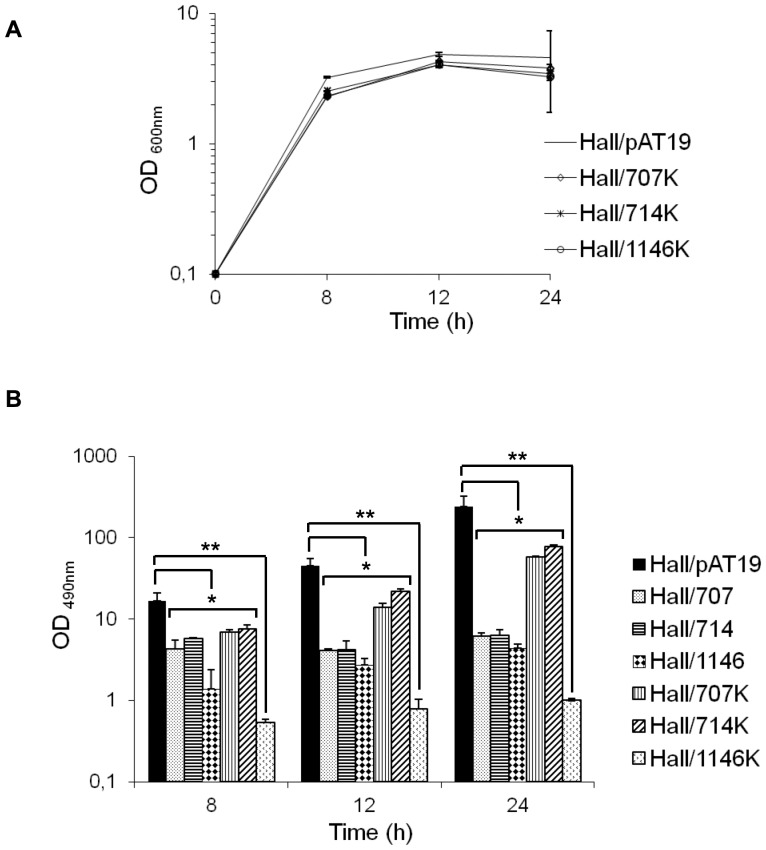
BoNT/A production in isogenic antisense strains targeting the SHK gene from three TCSs. (A) Growth kinetics of Hall/707K, Hall/714K, and Hall/1146K were similar to that of the control Hall/pAT19 strain as monitored by OD_600_ measurement. (B) BoNT/A production assayed by ELISA was significantly reduced in the culture supernatants of Hall/707K, Hall/714K, and to a higher extent of Hall/1146K. Data are mean values +/− SD from 3 independent experiments, *P<0.05 and **P<0.005.

In addition, the three isogenic antisense strains Hall/707, Hall/714, and Hall/1146 showed also a drastic reduction of ANTP accumulation in the culture supernatant including NTNH and HA34 (Fig. 5). This further supports the assumption that the three isogenic antisense strains were profoundly impaired in BoNT/A and ANTP synthesis. To confirm that the observed phenotype, i.e. decreased BoNT/A and ANTP production, did not result from spontaneous secondary mutations or antisense off-target effects, isogenic antisense strains were constructed that targeted the corresponding SHK gene. The isogenic antisense strains Hall/707K, Hall/714K, and Hall/1146K showed growth kinetics similar to those of the control strain Hall/pAT19 (Fig. 6A). BoNT/A production was reduced in the culture supernatants of the three isogenic antisense strains targeting the SHK gene compared to the control strain as monitored by ELISA (Fig. 6B). The decrease in BoNT/A production was lower in the strains Hall/707K and Hall/714K than in their counterparts Hall/707 and Hall/714 with antisense mRNA targeting the corresponding RR gene. Therefore, repression of the RR gene of some TCSs seems more efficient than that of the SHK gene. In contrast, for the TCS CLC_0661/CLC0663, targeted in the strains Hall/1146 and Hall/1146K, the inhibition of BoNT/A synthesis was significantly more pronounced when the SHK gene was repressed (Hall/1146K) compared to the the strain with the repressed RR gene (Hall/1146). This might indicate that this TCS particularly senses an environmental factor critical for BoNT/A synthesis in the applied culture conditions.

**Figure 7:**
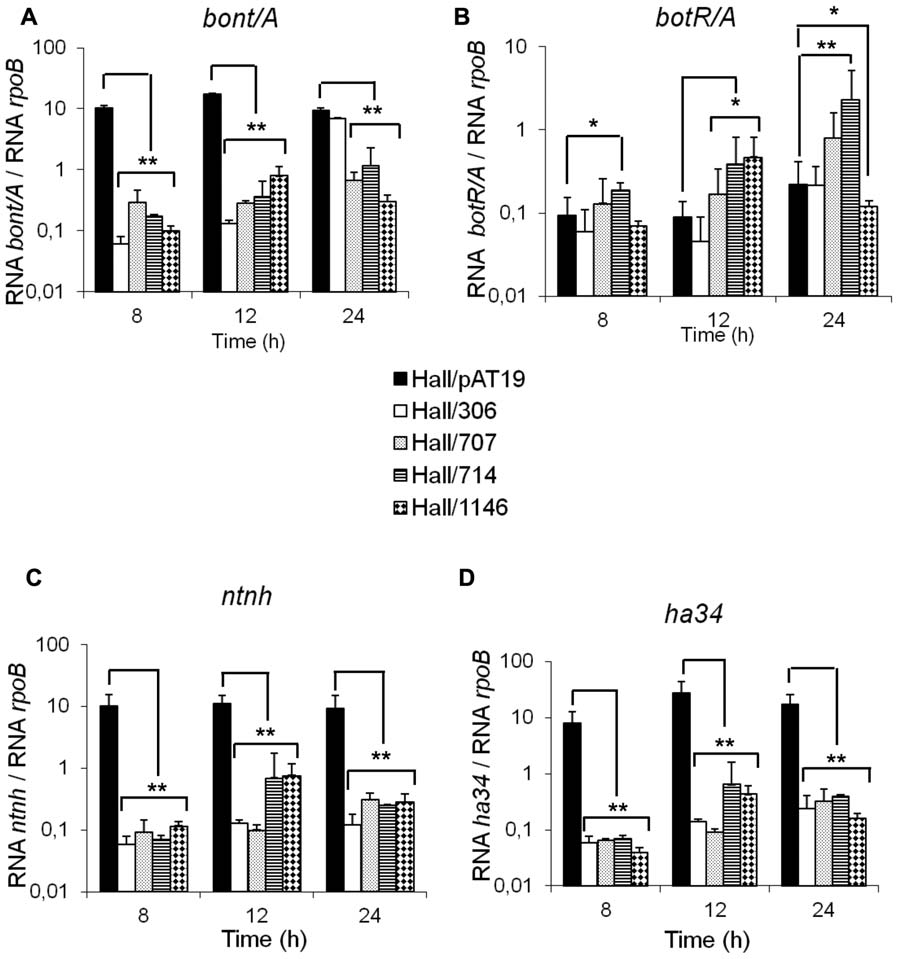
Kinetics of transcript levels of *bont/A, botR/A, ntnh*, and *ha34* genes in Hall/707, Hall/714, and Hall/1146 versus Hall/pAT19 and Hall/306. Transcript levels were quantified by qRT-PCR and normalized versus *rpoB* gene. Data are mean values +/− SD, from 9 independent experiments where *P<0.05 and **P<0.005.

To prove whether the repression of TCSs results in a decreased BoNT/A and ANTP production due to transcriptional repression, the expression of *bont/A* and *antp* genes was monitored by qRT-PCR. As shown in Fig. 7, the expression of *bont/A, ntnh, and ha34* genes was repressed in isogenic antisense strains Hall/707, Hall/714, and Hall/1146 similarly to that observed in the isogenic antisense strain repressed in *botR/A* (Hall/306). However, no alteration or even a slight increase in *botR/A* expression was observed in these isogenic antisense strains (Fig. 7B). In contrast to Hall/309, BoNT/A and ANTP synthesis (Fig. 3 and 5) was reduced in these isogenic antisense strains (Fig. 1). This suggests that the TCSs partially repressed in Hall/707, Hall/714, and Hall/1146 control the transcription of *bont/A* and *antp* genes independently of *botR/A*.

**Figure 8:**
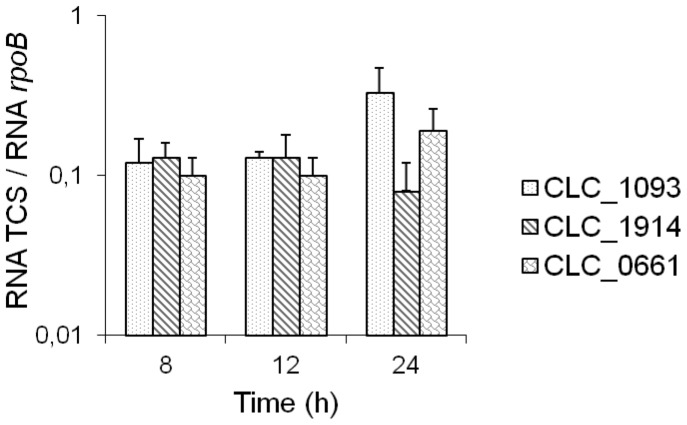
Kinetics of transcript levels of CLC_1093, CLC_1914, and CLC_0661 in Hall strain. Transcript levels were quantified by qRT-PCR and normalized versus *rpoB* gene. Data are mean values +/− SD, from 3 independent experiments.

The expression of the RR genes CLC_1093, CLC_1094, and CLC_0661 (partially repressed in strains Hall/707, Hall/714, and Hall/1146, respectively) was monitored and compared to *rpoB* gene expression (Fig. 8). The levels of expression were similar to that of *botR/A* (Fig. 2B). The three RR genes were expressed at similar levels throughout the exponential and early stationary growth phase concomitantly with *bont/A*, *ntnh* and *ha34* gene expression. Subsequently, at the beginning of the stationary phase (24 h), their expression seemed to diverge. However, the low differences in expression levels, slightly decreased for CLC_1914, and slightly increased for CLC_1093 and CLC_0661, were not statistically significant (Fig. 8).

## Discussion

BoNT synthesis in *C. botulinum* has been found to be a highly regulated process. *Antp* and *bont* genes are clustered in the botulinum locus and are transcribed as two polycistronic operons as evidenced by qRT-PCR or Northern blot analysis [25]–[27]. The *botR* gene, which is located in the botulinum locus in *C. botulinum* A, B, C, D, F and G plays a critical role in the regulation of BoNT and ANTP synthesis. Indeed, BotR/A has been demonstrated to be an alternative sigma factor controlling the transcription of the two operons of the botulinum locus at the transition phase from the exponential to the stationary growth phase [13], [14]. However, in *C. botulinum* type E, the transcription of *bont* and *antp* genes is also regulated during the transition phase, although *botR* or a homologous gene has not been identified in the genome of this *C. botulinum* type [14]. This strongly supports that *botR* is not the only regulatory gene controlling the expression of toxin gene in *C. botulinum*. Since at least 39 putative regulatory genes, distributed in TCS or orphan RR genes, have been identified in the genome of *C. botulinum* strain Hall, we have investigated their possible role in the control of BoNT/A synthesis.

A total of 34 Hall isogenic antisense strains have been generated with the antisense mRNA method targeting 29 regulatory genes predicted to be part of TCSs and 5 putative orphan RR genes. Among the 34 Hall isogenic antisense strains, 31 retained similar growth kinetics compared to the control strain, whereas 2 strains showed a more rapid growth and one isogenic antisense strain had a significantly delayed growth (Fig. 3B). BoNT/A was detected in a delayed manner in the culture supernatant of the isogenic antisense strain with a delayed growth kinetic (Hall/1001), but reached a similar OD than the control strain at 24 h, indicating that the repressed corresponding orphan RR (CLC_0632) is involved in global metabolism but does not directly control toxin synthesis. Interestingly, CLC_0632 shares sequence similarity with VirR (38% protein identity), which regulates the production of toxins in *C. perfringens* including prefringolysin, alpha-toxin and collagenase [17], [18], [28]. VirR belongs to a complex regulatory network controlling more than 147 genes such as genes for catalytic enzymes, transporters and energy metabolism and thus controlling multiple cellular functions [19], [20]. CLC_0632 does not have closer homologs (protein identity >60%) in other clostridia (Table 2). CLC_1105 also shows similarity to VirR (37% on protein level). However, Hall/1002 targeting CLC_1105 showed no alteration in growth kinetics and toxin production. The VirR homologs in *C. botulinum* Hall probably regulate basic functions but not specifically BoNT production.

The two isogenic antisense strains with a more rapid growth (Hall/1147 and Hall/1148) showed drastic changes in the bacterial cell wall or surface structure, which is probably the reason for the observed cell lysis. Reduced toxin levels were measured in the lyzed culture supernatant of both isogenic antisense strains reflecting lower BoNT synthesis rather than an impaired secretion. The corresponding RRs (CLC_0411 and CLC_3293), members of the OmpR family (Table 2), are probably part of regulatory cascades, which control multiple functions including bacterial surface polysaccharide synthesis and/or assembly and indirectly BoNT production. Besides the HATPase_c and the HisKA domains, the SHK CLC_0411 possesses a partial VicK domain (COG5002). Interestingly, VicK of *Streptococcus mutans* has been identified as a TCS sensor involved in the regulation of several virulence-associated genes affecting synthesis and adhesion to polysaccharides [30]. SHK CLC_3293 shows similarity to the BaeS family (COG0642) of sensors. Interestingly, the BaeS sensor in *E. coli* is involved in envelope stress response [31]. Taken together, it can be hypothesized that CLC_0410/CLC_0411 and CLC_3294/CLC_3293 are TCSs involved in regulating cell surface properties, e.g. surface polysaccharide synthesis and integrity.

Interestingly, a quorum sensing system related to that of *Staphylococcus aureus* and consisting of two *agr* loci has been identified in the group I of *C. botulinum* strains, which controls both sporulation and BoNT production. Each *agr* locus seems to have a specific function, *agr*-1 regulating sporulation and *agr*-2 regulating BoNT synthesis [29]. We have also identified homologous genes to *agrA* and *agrC* from *S. aureus* in the Hall genome. The isogenic antisense strain Hall/651 targeting the *agrA* homolog was not impaired in BoNT production. Thus, the quorum sensing-dependent regulation pathway and its effects on the control of toxin production in *C. botulinum* remain to be defined.

**Figure 9:**
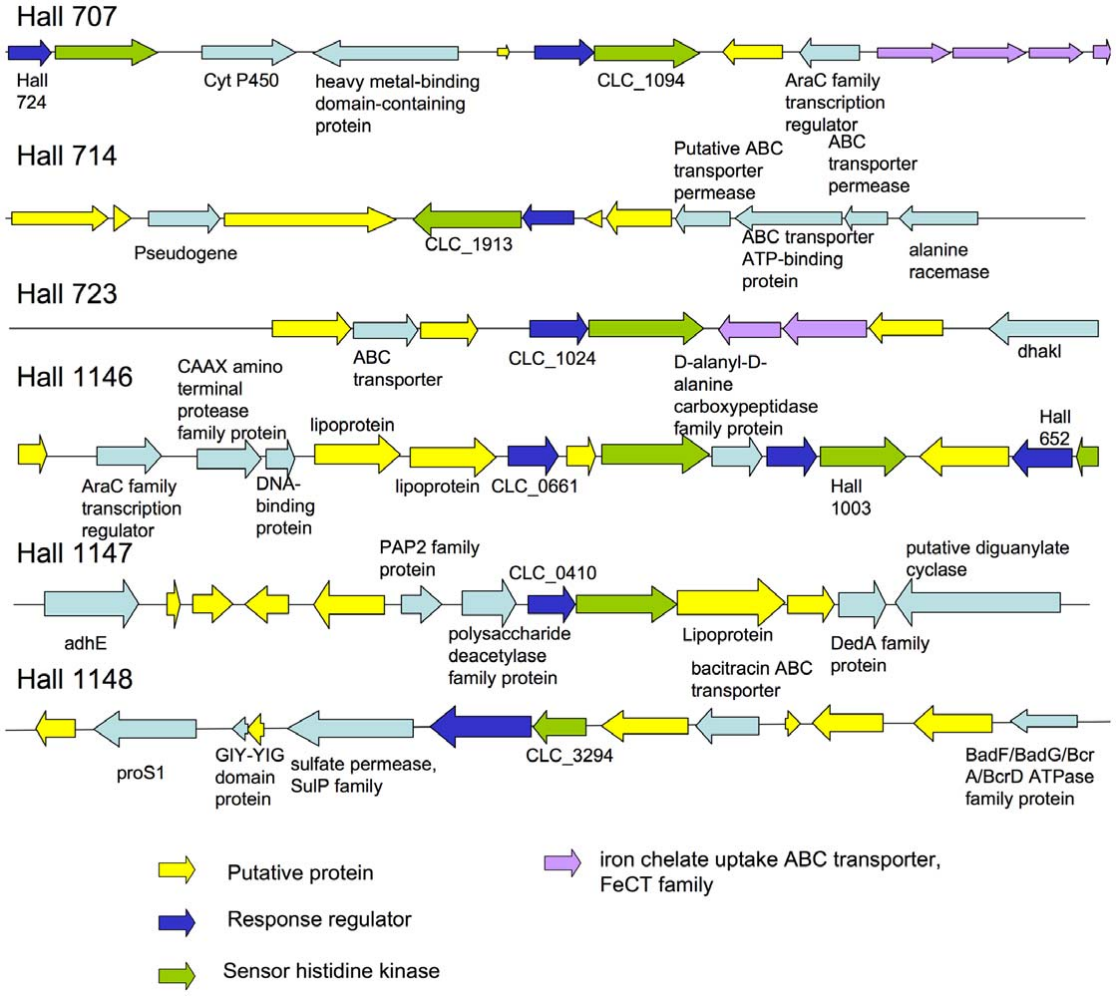
Genetic environment of the TCS genes involved in the regulation of toxin synthesis in strain Hall.

We showed that three TCSs, CLC_1093/CLC_1094, CLC_1914/CLC_1913 and CLC_0661/CLC_0663 (were involved in the regulation of e BoNT/A and ANTP production. BoNT/A, NTNH and HA34 levels in the culture supernatants of the corresponding isogenic antisense strainsHall/707, Hall/707K, Hall/714, Hall/714K, Hall/1146, Hall/1146K were repressed similarly to that observed in the *botR/A* isogenic antisense strain (Hall/306). Expression of *bont/A, ntnh*, and *ha34* genes was reduced throughout the 24 h growth period, as also observed in Hall/306. However, *botR/A* expression was not affected or even slightly increased in the three Hall isogenic antisense strains. These results argue that the corresponding TCSs, CLC_1093/CLC_1094, CLC_1914/CLC_1913 and CLC_0661/CLC_0663, control, directly or indirectly, the expression of the botulinum locus genes independently of *botR/A*. Other regulatory genes, notably from the AraC family, and genes encoding for transporters are distributed in the vicinity of these TCSs (Fig. 9). This suggests that the TCSs could be part of a complex regulatory network in response to external signals. However, the TCS might interact with other genes, which are distantly located on the genome. The biological role of the TCS CLC_0661/CLC_0663 remains to be investigated. The TCS is homologous to TCSs of the PhoP/PhoR family involved in, but not restricted to, sensing and reacting to phosphate starvation. It is homologous to CTC00411/CTC00412 of *C. tetani* (65% and 53% protein identity, respectively), and CPE2098/CPE2099 of *C. perfringens* (50 and 40% identity, respectively). In the latter organism, the system has been designated VirI/VirJ, and it has been described as “a novel two-component regulatory system involved in the shutdown of extracellular toxin production in *C. perfringens*”, although further data is not available (EMBL/GenBank/DDBJ databases: BAA78773.1 and BAA78774.1).

Albeit *C. botulinum* and *C. tetani* synthesize related neurotoxins, a specific regulatory network based on TCSs seems to control BoNT production in *C. botulinum* type A. The 5 orphan regulatory genes tested in strain Hall (Table 2) seem not to be involved in the regulation of the botulinum locus genes. Interestingly, the 5 TCSs, which directly or indirectly are involved in the regulation of toxin synthesis in strain Hall, are conserved in the known genomes of *C. botulinum* from group I including the subtypes A2, A3, Ba4, B1 and F, but not in the genomes of group II strains such as *C. botulinum* type E or group IV (*C. botulinum* C and D). These results suggest that group I *C. botulinum* strains might share a regulatory network distinct from that in the other *C. botulinum* groups.

This raises the question about the external signals governing the toxin production via TCS regulation. The environmental factors controlling toxin gene expression in *C. botulinum* are still poorly known. Carbon dioxide has been reported to stimulate toxin gene expression and toxin formation in non-proteolytic *C. botulinum* strains type B and E despite a growth rate reduction [32], [33], in contrast to proteolytic *C. botulinum* type A, which seems insensitive to carbon dioxide [34], [35]. High temperature did not control the transcription of *bont* gene but influences the stability of the toxin in *C. botulinum* A [14]. A better understanding of the regulation of toxin synthesis in *C. botulinum* would permit the development of novel strategies to prevent botulism by counteracting the toxin production in food and/or in the digestive tract.

In summary, BoNT/A and ANTP synthesis in *C. botulinum* strain Hall seems to be under the control of a complex regulatory network. In addition to the alternative sigma factor BotR/A, which regulates *bont/A* and *antp* transcription at the transition phase between the exponential and stationary growth, we have found that at least three TCSs also control BoNT/A and ANTP synthesis at the transcriptional level independently of BotR/A. Two other TCSs seem to retain various pleiotropic effects including the control of BoNT/A synthesis as well as the regulation of bacterial cell wall or cell surface synthesis and/or assembly and granule accumulation in the cytoplasm. Further investigations of the regulatory network controlling toxin production in *C. botulinum* would allow the identification of environmental factors triggering BoNT/A synthesis.

## Materials and Methods

### Bacterial strains and culture conditions

The recombinant strains are presented in figure 1. pat19 vector was used for the construction of the recombinant ARNm antisense strains [12]. *Escherichia coli* strains were grown in Luria-Bertani (LB) broth and *Clostridium botulinum* strains in TGY broth (pH 7,5) containing trypticase (Trypticase-Glucose Yeast Peptone BBL, BD Biosciences; 30 g/L), yeast extract (Bacto Yeast Extract, BD Biosciences; 20 g/L), glucose (5 g/L) and cystein, HCl (0,5 g/L) under anaerobic conditions (N2/CO2/H2; 90 : 5 : 5, vol/vol. at 37°C). When necessary, erythromycin was added to culture media at 20 µg/ml for *C. botulinum* and 300 µg/ml for *E.coli*.

Kinetic experiments of *C. botulinum* recombinant strains were performed by incubating 10 ml TGY inoculated with 0.3 to 0.6 ml of an overnight preculture to obtain a starting culture of 0.1 OD_600_. Bacterial growth was monitored as OD_600_ every two hours until 12 h, then at 24 and 48 hours of culture for the control strains and at 8, 12 and 24 hours for isogenic antisense strains. Bacterial counting was performed in agarose TGY medium supplemented with erythromycin (5 µg/ml). Serial ten-fold dilutions of cultures were plated on agarose-TGY (0.1 ml on each plate, 3 plates for each dilution) and incubated at 37°C in anaerobic chamber.

### Construction of vectors encoding antisens mRNA for the different two-component systems

A DNA fragment of each TCS gene studied containing the Ribosome Binding Site (RBS) region was amplified by PCR and inserted in reverse orientation into pAT19 as already described in [12].

### Botulinum neurotoxin and associated protein assay

At 2, 4, 6, 8, 10, 12, 24 and 48 hours culture, 10 ml of culture were removed. The cells were harvested at 15000 rpm for 5 minutes, and the supernatants were removed and stored at −20°C. The pellets were stored at −80°C for quantitative RT-PCR analysis.

The toxin content of the supernatants was assessed using an enzyme-linked immunosorbent assay (ELISA). Ninety-six-well microtitre plates (Maxisorp, Nunc Roskilde, Denmark) were sensibilized with 100 µl of monoclonal anti Hc BoNT/A antibody Mab G14-3 raised against HcBoNT/A1 as previously described [36] (5 µg/ml in carbonate buffer 0.05 M pH 9.6) and incubated at 4°C over night. Plates were washed 3 times with PBS-Tween (0.1%) (PBST) with an automatic plate washer (Bio-Tek, Washer 120) and saturated with 100 µl of BSA (2% in carbonate buffer) during 30 min under agitation. Three washes were performed and 100 µl of supernatant were added and dilute by half with PBST. After 30 minutes of incubation under agitation, three washes were performed, and 100 µl of polyclonal rabbit antibody raised against HcBoNT/A1 diluted at 1/1600 in PBST, and incubated for an hour under agitation. Three new washes were performed and 100 µl of goat peroxydase-linked antibody anti-rabbit Ig (111-035-006, Jackson Immunoresearch) diluted at 1/5000 in PBST was added and plate were incubated 1 h under agitation. After the last three washes, detection of toxin was performed by adding 100 µl of OPD in citrate buffer (0.05 M, 0.06% H2O2, 1 mg/mL of ortho-phenylen-diamin, Sigma) and incubated seven minutes. Optical density was measured at 490 nm with a spectrophotometer microplate reader (Biorad, model 680).

The amounts of associated protein HA34 and NTNH in the supernatants were assessed using a Western blot assay. Total proteins of 800 µl of a 12 hours culture supernatant were precipitated with sulfuric acid (pH 3.5) and harvested 5 minutes at 15000 rpm. Pellets were resuspended in 200 µL of Laemmli buffer and 15 µl were loaded on a 10% SDS PAGE. The antibodies used and the immunoblotting procedure were the same as previously described [12]. Bound antibodies were detected using an anti rabbit IgG antibody coupled to alkaline phosphatase, ECF™ Substrate for Western Blotting supplied by Ge Healthcare and a Storm fluorescent scanning. Western blots were quantified using Image J software.

### Mouse bioassay

In vivo mouse lethality tests were performed for each isogenic antisense strains showing a manifest decrease in toxin production by ELISA compared to the control strain. Ten-fold serial dilution of 24 h culture supernatants in 50 mM phosphate buffer pH 6.5 containing 1% gelatin (PB-G) were done. 0.5 mL was injected intraperitoneally (i.p) into Swiss mice weighing 20–22 g (Charles River). Mice were observed and any deaths recorded every day for 4 days.

### Ethics statement

All experiments were performed in accordance with French and European Community guidelines for laboratory animal handling. The protocols of experiments were approved by the Pasteur Institut (Agreement of laboratory animal use n° 75–279).

### Total RNA extraction, Reverse Transcription and Quantitative real-time PCR assay

A phenol-chloroform extraction of total RNA was performed from pellets of each recombinant strains at 8, 12 and 24 hours, and at each kinetic point for the Pat19, Hall306 and Hall309 strains. Cells were mechanically broken by shaking with a FastPrep apparatus (MP Biomedicals) and RNA was extracted with Trizol reagent, and chloroform-isoamylcalcohol. The extracted RNAs were precipitated with isopropanol and the pellet was resuspended in a 10 mM Tris pH = 8, 0.1 mM EDTA buffer. A DNAse treatment with TURBO DNase (Ambion) was performed on each extract following the manufacturer's instructions. The total RNA quantity was measured using a NanoDrop ND-100 Spectrophotometer and cDNA were synthesized from 5 µg of total RNA with M-MLV Reverse Tanscriptase kit (Invitrogen) according to manufacturer's instructions. Hexamer oligonucleotide primers (pdN6 5 µg/µL), deoxynucleoside triphosphates (dNTP; 10 mM each), and RNase OUT™ Recombinant Ribonuclease Inhibitor are provided from Roche, Amersham Biosciences and Invitogen respectively. Each extract was analysed at least once with Agilent 2100 Biolanalyser with RNA 6000 Nano Reagents & Supplies (Roche).

Reverse-transcribed cDNA samples (30 ng) were subjected to PCR amplification in 24 µl ready-to-use iQ SYBR Green Supermix (Bio- Rad, 2X; 1,25 U iTaq DNA polymerase, 0,4 mM each dNTP, 6 mM MgCl2, 20 nM fluorescein, SYBR Green I) containing 0,5 mM each primer. The reactions were cycled in an iQ iCycler apparatus (Bio-Rad) using the following parameters: Taq polymerase activation at 95°C for 3 minutes then 39 cycles at 95°C for 10 s, 51°C for 30 s for *ha34* and *botR/A* genes and 65°C for *rpoB ntnh* and *bont/A* genes for the annealing and the extension with fluorescence measurement at the end. A temperature gradient was performed for each primer pair with serial 10-fold dilutions of Hall chromosomal DNA (1.5 pg to 150 ng) to determine the optimal annealing temperature giving the maximal PCR efficiency (supplementary Table 1). Each cDNA sample was PCR amplified in parallel to serially diluted Hall chromosomal DNA. cDNA quantity of gene was normalized to the quantity of cDNA of the *rpoB* gene. The relative cDNA quantity of each sample was determined with threshold cycle [ΔΔCT] method (Analysis of Relative Gene Expression Data Using Real- Time Quantitative PCR and the 2ΔΔCT method) [37].

The identity and specificity of PCR products were verified by high resolution agarose gel electrophoresis and a single PCR product migrating at the expected size was observed for each primer pair. In addition, an iQ iCycler melting curve (55°C to 95°C with a heating rate of 0, 5°C per 10 s and continuous fluorescence measurement) confirmed the presence of a unique peak for the negative first derivative of the temperature versus fluorescence plotted against temperature (melt curve) with single product-specific melting temperatures (Tm).

### Transmission electron microscopy

All bacterial strains were pelleted using a tabletop centrifuge and afterwards resuspended in a small amount of medium. The concentrated suspension was taken up in a cellulose cappillary tube (Leica microsystems, Vienna, Austria). The tube was cut into closed pieces not longer than 2 mm with a modified scalpel [38] and placed into planchettes of 200 µm depth fill with 1-hexadecen. Cryo-immobilisation was done using a HPM 010 high-pressure freezer (BalTec, now Abra Fluid AG, Widnau, Switzerland) and samples transferred under liquid nitrogen for freeze substitution in 1% osmium tetroxide (Merck, Darmstadt, Germany) containing 0.5% glutaraldehyde (EMS, Hatfield, USA) and 0.2% uranyl acetate (SPI, West Chester, USA) in acetone containing 2% water in an AFS (Leica microsystems, Vienna, Austria). Substitution was performed for 48 hours at −90°C, samples were warmed up (2°C/hour) to −30°C (8 hours) and then warmed up to 0°C before removal of the substitution medium and embedding in EPON. After heat polymerization thin sections were cut with an Ultracut UCT microtome (Leica Microsystems, Vienna, Austria). Sections were collected on 200 mesh formvar coated copper grids. After poststaining with 4% uranyl acetate and Reynold's lead citrate, the sections were observed with a Jeol 1010 at 80 kV and equipped with a KeenView camera (Olympus, Soft imaging systems, Münster, Germany).

### Statistics

Values throughout are expressed as means +/− standard deviation (SD). Differences in the different isogenic antisense strains were assessed using unpaired Student's t-test where statistical significance is assumed for *P<0.05 and **P<0.005.

## Supporting Information

Table S1 Optimization of quantitative PCR amplification. PCR efficiencies were calculated from a standard curve performed with a serial 10-fold dilution of chromosomal Hall DNA and a temperature gradient ranging from 51 to 68°C for each primer pair targeting *bont/A, ntnh, rpob, botR/A*, and *ha34* genes. Annealing temperature of 51°C was used in qRT-PCR for *ha34* and *botR/A* genes, and 65°C for *bont/A, ntnh*, and *rpob* genes. These annealing temperatures yielded a PCR efficiency close of 100% in the largest range of DNA detection.
(DOC)
