# Correction: Two-Component Systems Are Involved in the Regulation of Botulinum Neurotoxin Synthesis in *Clostridium botulinum* Type A Strain Hall

**DOI:** 10.1371/annotation/c61c1b9e-b406-4057-99c6-ff84d67869bf

**Published:** 2012-10-26

**Authors:** Chloé Connan, Holger Brüggemann, Christelle Mazuet, Stéphanie Raffestin, Nadège Cayet, Michel R. Popoff

The second author's name was spelled incorrectly. The correct name is: Holger Brüggemann.

The correct citation is: Connan C, Brüggemann H, Mazuet C, Raffestin S, Cayet N, et al. (2012) Two-Component Systems Are Involved in the Regulation of Botulinum Neurotoxin Synthesis in *Clostridium botulinum* Type A Strain Hall. PLoS ONE 7(7): e41848. doi:10.1371/journal.pone.0041848

