# Supplementary material for: Two-Component Systems Are Involved in the Regulation of Botulinum Neurotoxin Synthesis in Clostridium botulinum Type A Strain Hall
Source: PLoS One. 2012 Jul 26;7(7):e41848. doi: 10.1371/journal.pone.0041848 (PMC3406050; doi:10.1371/journal.pone.0041848)
Supplement: Table S1 — Optimization of quantitative PCR amplification. PCR efficiencies were calculated from a standard curve performed with a serial 10-fold dilution of chromosomal Hall DNA and a temperature gradient ranging from 51 to 68°C for each primer pair targeting bont/A, ntnh, rpob, botR/A, and ha34 genes. Annealing temperature of 51°C was used in qRT-PCR for ha34 and botR/A genes, and 65°C for bont/A, ntnh, and rpob genes. These annealing temperatures yielded a PCR efficiency close of 100% in the largest range of DNA detection. (DOC) [file pone.0041848.s001.doc]

| *bont/A* | | | | |
| --- | --- | --- | --- | --- |
| T (°C) | PCR efficiency (%) | *R^2^* | Slope | Range of detected DNA |
| 68 | 95 | 0.997 | 3.44 | 1.5 pg – 150 ng |
| 67 | 94.1 | 0.999 | 3.47 | 1.5 pg – 150 ng |
| 65.1 | 99.6 | 0.998 | 3.33 | 1.5 pg – 150 ng |
| 61.7 | 87.1 | 0.999 | 3.67 | 1.5 pg – 150 ng |
| 57.6 | 82.6 | 0.999 | 3.82 | 1.5 pg – 150 ng |
| 54.4 | 77.6 | 0.999 | 4 | 1.5 pg – 150 ng |
| 52.1 | 79.8 | 0.998 | 3.92 | 1.5 pg – 150 ng |
| 51 | 74.5 | 0.999 | 4.13 | 1.5 pg – 150 ng |

| *ntnh* | | | | |
| --- | --- | --- | --- | --- |
| T (°C) | PCR efficiency (%) | *R^2^* | Slope | Range of detected DNA |
| 68 | 83.2 | 0.998 | 3.8 | 1.5 pg – 150 ng |
| 67 | 89.3 | 0.996 | 3.61 | 1.5 pg – 150 ng |
| 65.1 | 96.5 | 0.999 | 3.41 | 1.5 pg – 150 ng |
| 61.7 | 88.4 | 0.999 | 3.63 | 1.5 pg – 150 ng |
| 57.6 | 79.9 | 0.994 | 3.92 | 1.5 pg – 150 ng |
| 54.4 | 70.3 | 0.992 | 4.32 | 15 pg – 150 ng |
| 52.1 | 99.6 | 0.007 | 0.42 | 15 pg – 150 ng |
| 51 | 174.2 | 0.462 | 2.28 | 15 pg – 150 ng |

| *rpob* | | | | |
| --- | --- | --- | --- | --- |
| T (°C) | PCR efficiency (%) | *R^2^* | Slope | Range of detected DNA |
| 68 | 100 | 0.993 | 3.32 | 1.5 pg – 150 ng |
| 67 | 99 | 0.999 | 3.34 | 1.5 pg – 150 ng |
| 65.1 | 105.1 | 0.996 | 3.2 | 1.5 pg – 150 ng |
| 61.7 | 94.6 | 0.998 | 3.15 | 1.5 pg – 150 ng |
| 57.6 | 89.1 | 0.998 | 3.61 | 1.5 pg – 150 ng |
| 54.4 | 93.5 | 0.993 | 3.48 | 1.5 pg – 150 ng |
| 52.1 | 87.9 | 0.999 | 3.65 | 1.5 pg – 150 ng |
| 51 | 79.2 | 0.999 | 3.94 | 1.5 pg – 150 ng |

| *botR/A* | | | | |
| --- | --- | --- | --- | --- |
| T (°C) | PCR efficiency (%) | *R^2^* | Slope | Range of detected DNA |
| 68 | 159.5 | 0.956 | 2.41 | 15ng -- 150ng |
| 67 | 106.8 | 1 | 3.16 | 15ng -- 150ng |
| 65.1 | 155.6 | 0.872 | 2.45 | 150pg – 150ng |
| 61.7 | 99.1 | 0.997 | 3.34 | 150pg -- 150ng |
| 57.6 | 108.1 | 0.983 | 3.14 | 1.5 pg – 150 ng |
| 54.4 | 96.2 | 0.996 | 3.41 | 1.5 pg – 150 ng |
| 52.1 | 95.8 | 0.988 | 3.42 | 1.5 pg – 150 ng |
| 51 | 95.6 | 0.981 | 3.43 | 1.5 pg – 150 ng |

| *ha34* | | | | |
| --- | --- | --- | --- | --- |
| T (°C) | PCR efficiency (%) | *R^2^* | Slope | Range of detected DNA |
| 68 | 123.7 | 0.996 | 2.86 | 1.5ng -- 150ng |
| 67 | 100.9 | 0.999 | 3.3 | 1.5ng -- 150ng |
| 65.1 | 113.5 | 0.998 | 3.03 | 150pg -- 150ng |
| 61.7 | 120.5 | 0.976 | 2.91 | 150pg -- 150ng |
| 57.6 | 114.1 | 0.973 | 3.02 | 1.5 pg – 150 ng |
| 54.4 | 98.2 | 0.814 | 3.36 | 1.5 pg – 150 ng |
| 52.1 | 107.9 | 0.974 | 3.14 | 1.5 pg – 150 ng |
| 51 | 105.3 | 0.972 | 3.2 | 1.5 pg – 150 ng |

| *CLC_1093* | | | | |
| --- | --- | --- | --- | --- |
| T (°C) | PCR efficiency (%) | *R^2^* | Slope | Range of detected DNA |
| 68 | 61.7 | 0.977 | 4.82 | 150pg -- 150ng |
| 67 | 82.9 | 0.993 | 3.81 | 1.5 pg – 150 ng |
| 65.1 | 78.6 | 0.993 | 3.97 | 15 pg – 150 ng |
| 61.7 | 79.6 | 0.990 | 3.93 | 15 pg – 150 ng |
| 57.6 | 78.9 | 0.993 | 3.96 | 15 pg – 150 ng |
| 54.4 | 81.9 | 0.988 | 3.84 | 15 pg – 150 ng |
| 52.1 | 88.7 | 0.962 | 3.62 | 1.5 pg – 150 ng |
| 51 | 81.7 | 0.909 | 3.85 | 1.5 pg – 150 ng |

| *CLC_1914* | | | | |
| --- | --- | --- | --- | --- |
| T (°C) | PCR efficiency (%) | *R^2^* | Slope | Range of detected DNA |
| 68 | 150,2 | 0,889 | 2,51 | 1.5 pg – 150 ng |
| 67 | 136,7 | 0,927 | 2,67 | 1.5 pg – 150 ng |
| 65.1 | 126 | 0,948 | 2,82 | 1.5 pg – 150 ng |
| 61.7 | 126,2 | 0,933 | 2,82 | 1.5 pg – 150 ng |
| 57.6 | 129,6 | 0,908 | 2,77 | 1.5 pg – 150 ng |
| 54.4 | 133,1 | 0,889 | 2,72 | 1.5 pg – 150 ng |
| 52.1 | 143,7 | 0,895 | 2,59 | 1.5 pg – 150 ng |
| 51 | 150,7 | 0,871 | 2,50 | 1.5 pg – 150 ng |

| *CLC_0661* | | | | |
| --- | --- | --- | --- | --- |
| T (°C) | PCR efficiency (%) | *R^2^* | Slope | Range of detected DNA |
| 68 | 86 | 0,976 | 3,71 | 15 pg – 150 ng |
| 67 | 91,9 | 0,991 | 3,53 | 15 pg – 150 ng |
| 65.1 | 96,2 | 0,984 | 3,42 | 15 pg – 150 ng |
| 61.7 | 88,3 | 0,993 | 3,63 | 1.5 pg – 150 ng |
| 57.6 | 97,3 | 0,978 | 3,39 | 1.5 pg – 150 ng |
| 54.4 | 100,3 | 0,965 | 3,32 | 1.5 pg – 150 ng |
| 52.1 | 102,6 | 0,970 | 3,26 | 1.5 pg – 150 ng |
| 51 | 99,6 | 0,963 | 3,33 | 1.5 pg – 150 ng |

**Supplementary Table 1**
